# Neural network properties of hydrophilic polymers as a key for development of the general theory of evolution

**DOI:** 10.1098/rsos.242149

**Published:** 2025-04-23

**Authors:** Sherniyaz Kabdushev, Oleg Gabrielyan, Eldar Kopishev, Ibragim Suleimenov

**Affiliations:** ^1^ Department of Chemistry and Technology of Organic Materials, Polymers and Natural Compounds, Al-Farabi Kazakh National University, Almaty, Kazakhstan; ^2^ VI Vernadsky Crimean Federal University, Simferopol, Ukraine; ^3^ Department of Chemistry, L.N. Gumilyov Eurasian National University, Astana, Kazakhstan; ^4^ Bukhara State University, Bukhara, Uzbekistan; ^5^ National Engineering Academy of the Republic of Kazakhstan, Almaty, Kazakhstan

**Keywords:** neural networks, coevolution, information processing systems, dialectics, hydrophilic polymers

## Abstract

The analysis of the existing literature demonstrates that in order to address the fundamental challenges associated with the origin of life, it is essential to consider this problem from a comprehensive perspective, specifically from the vantage point of the general theory of evolution of complex systems. From these positions, life should be regarded as a distinctive instance of an information storage and processing system that emerges naturally. Evolutionary processes should be examined from the vantage point of the coevolution of material and informational components, which has not been sufficiently emphasized hitherto. It is shown that a specific example in this respect is analogues of neural networks spontaneously formed in solutions of some hydrophilic polymers. Such systems lead to the formation of non-trivial information objects. A wide range of other examples is considered, proving that the processes occurring with the participation of hydrophilic polymers should be interpreted, among other things, from the point of view of formation of information objects, which, under certain conditions, influence the processes occurring at the molecular and supramolecular level. It is shown that it is reasonable to use the tools of classical dialectics to solve such fundamental problems as that of the origin of life.

## 1. Introduction

The mechanisms of pre-biological evolution are far from being fully revealed; moreover, the question remains open as to how far the evolutionary theories originally developed in biology are applicable to describe the processes that led to the emergence of life [1]. The cited report emphasizes that this question has, among other things, a philosophical aspect.

It is impossible to disagree with this statement. It would not be a great exaggeration to say that evolutionary theory (understood in the broadest sense of the term) de facto claims the status of a general scientific theory, i.e. applicable to the description of phenomena of any nature (from chemical to social). Consequently, it cannot but require, at least, additional justifications of a philosophical nature.

**Figure 1.**
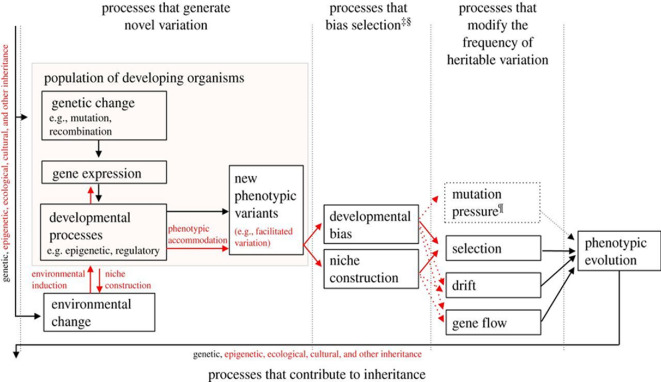
The structure of the extended evolutionary synthesis [3].

There are substantial grounds for considering evolutionary theory built on the ideas of Darwinism as a general scientific theory (or as the basis of a general scientific theory). As noted in [1], classical Darwinism has undergone a rather complex path of development, having absorbed in the early twentieth century the achievements of Mendelian and population genetics (Modern Synthesis, [2]), and further, ecology and a number of other disciplines, resulting in the emergence of studies united by the umbrella name of the Extended Evolutionary Synthesis [3,4] (figure 1).

The methodological attractiveness of evolutionary theory contributed to the penetration of its ideas into related disciplines. There are numerous attempts to use evolutionary theory in economics, psychology, social sciences and many other disciplines [5–7].

One of the brightest examples in this respect is Social Darwinism [8]. It has been repeatedly criticized [9,10], but this does not invalidate the above-mentioned fact that evolutionary theory de facto claims a general scientific status in many respects. This is also evidenced by the emergence of a purely philosophical concept: global evolutionism [11,12].

One of the key problems closely related to the question of the general scientific status of evolutionary theory is obviously the problem of the origin of life. This problem, as it is rightly noted in [1], becomes the very problem on the example of which the adequacy of various theories of evolution, including those claiming to be all-encompassing, is tested.

Moreover, the authors [1] justifiably emphasize the importance of philosophical understanding not only of the problem of the origin of life itself, but also of philosophical understanding of the basic provisions of evolutionary theory.

In this connection, however, it makes sense to draw attention to the following circumstance. In works in which evolutionary theory is considered from the most general positions [13,14], the term ‘evolution’ is often de facto synonymous with the term ‘development’ in its philosophical sense.

Let us recall that the category of development is one of the fundamental ones for classical philosophy, especially for classical dialectics going back to Hegel [15]. Consequently, at a minimum, the question arises as to how the understanding of evolution—in the broadest possible sense of the term—relates to the philosophical category of development.

In this review, we demonstrate that the appeal to dialectics in relation to the problem of the origin of life (more broadly, the mechanisms of evolution of complex systems) is by no means a mind game. Dialectics (not necessarily materialist) is the only form of philosophical understanding of reality purposefully designed to deal with contradictions [16–18].

We intend to show that the contradictions arising in attempts to reveal the mechanisms of prebiological evolution, as reflected in the current literature, including the review [1], reflect, first of all, the contradictory nature of evolutionary processes. As it follows from the materials of this review, contradictoriness is a fundamental property of the processes under consideration, ignoring which leads to a one-sided view of things.

The term ‘contradictions’ is also used here in the sense of classical dialectics, which treats the contradictory nature of phenomena inherent in reality as a source of development [16–18]. Recall that according to classical dialectics, the category of contradiction is most closely related to Hegel’s law of unity and struggle of opposites [16–18]. We will not dwell in detail on the arguments reflected in Hegel’s Science of Logic, but we shall cite his statement, which is difficult to disagree with: ‘Contradiction is the root of all movement and vitality: only insofar as something has contradiction within itself does it move, possess impulse, and activity’.

Reference to the aforementioned philosophical law when analysing existing views on the problem of the origin of life also seems reasonable. Namely, in this review, we will demonstrate that none of the existing perspectives can fully elucidate the mechanisms underlying the origin of life (or, more broadly, the evolution of complex systems). A dialectical synthesis is required—one that considers the unity of opposites—where contradictions are acknowledged as an intrinsic aspect of reality, governing its non-trivial behaviour. The justification for this assertion is provided on the basis of classical dialectics.

To concretize the system of evidence, a specific class of substances—hydrophilic polymers—is considered. The selection of this particular class of chemical compounds is based on the following considerations.

Firstly, research in this field has for many decades been largely oriented on analogies with objects of a biological nature [19,20]. More precisely, synthetic polymers have been and are being used, among other things, to establish regularities of behaviour of biological analogues, in particular, such hydrophilic biopolymers as DNA and RNA [21,22].

Second, it is the question of the probability of the emergence of highly ordered hydrophilic DNA and RNA biopolymers that has been and is regarded as one of the ‘stumbling blocks’ for evolutionary theories of the origin of life [23,24], which goes back to the so-called ‘Jenkins nightmare’ [25].

Thirdly, and this is proved in the materials of this review, in order to find an answer to the question about the mechanisms of the origin of life, it should be put more broadly. Specifically, Life can and should be considered, including from the point of view of information theory, which was emphasized, in particular, in [26]. An obvious example: reproduction of viruses as an object intermediate between living and inert matter meets the solution of only one task—preservation of genetic information by reproducing it. Accordingly, the question about the origin of life in a generalized form is formulated as follows.

What are the mechanisms for the spontaneous emergence of systems capable of storing and processing information?

This formulation of the question allows us to look at the role of research in the field of physical chemistry of hydrophilic polymers from a somewhat unexpected perspective. Indeed, numerous systems that can be considered as information processing systems have already been obtained on the basis of such polymers. The simplest example here is drug delivery systems that release active substances according to a predetermined programme [27,28]. There are also numerous examples of advanced applications of polymers in information technologies. Thus, attempts have been made to implement optical neural networks based on this approach [29]. The use of hydrophilic polymers for the development of sensor technology was proposed in [30,31]. Compounds of the considered type can also serve as the basis for the creation of metamaterials [32–34], which exhibit unique capabilities for signal processing in both the optical and radio frequency ranges due to their negative refractive index [35,36]. Cross-linked networks derived from hydrophilic polymers enable the realization of neuromorphic materials [37,38], which constitute a physical implementation of neural networks. There are solid grounds to believe [39] that such materials, which are currently the subject of active research [40], may ultimately facilitate the development of novel types of computing systems, including those free from the inherent limitations of semiconductor-based computing technology (such as the necessity for data exchange between computational units and memory blocks [40]).

Neuromorphic materials are typically created artificially [39]. However, there also exist physicochemical systems in which analogues of neural networks form spontaneously [41,42]. The fact seems to be quite remarkable, since nowadays the problem of the origin of life is more and more often considered precisely in terms of analogies with neural networks [43–45]. As is known, many information processing systems are now realized on the basis of neural networks, primarily artificial intelligence [46,47]. Consequently, the processes of self-organization occurring in polymer solutions, which have also been studied in numerous works, for example in [48–50], can be viewed from a purely informational point of view.

In this review, on the basis of literature data, it is argued that the consideration of the processes occurring in hydrophilic polymer solutions on the basis of the information point of view, at least, creates additional prerequisites for the construction of a general theory of evolution, including for establishing the true mechanisms that led to the emergence of Life.

The analysis presented in the review allows us to make the following statements:

– The question of the origin of life cannot be fully resolved without the development of a comprehensive evolutionary theory.
– The creation of a general evolutionary theory necessitates the most intimate interdisciplinary collaboration between biology, the chemistry of high-molecular compounds, information theory and other sciences, including philosophy (more specifically, dialectics).
– A general evolutionary theory can only be created using the tools of dialectics, as this is the only tool designed to make sense of contradictions. Contradiction, understood in the philosophical sense, is one of the basic attributes of evolution.

This review is distinctive in that it endeavours, on the basis of evidence already documented in the literature, to establish a methodological framework for the construction of a general theory of evolution.

The system of evidence is constructed based on the following theses:

– From a general methodological standpoint, it is evident that the origin of life cannot be fully elucidated without the construction of a comprehensive evolutionary theory. Conversely, the resolution of the problem of the origin of life (the most complex phenomenon considered by evolutionary theory) will entail, at the very least, the establishment of the prerequisites for the theoretical description of evolutionary processes of any type [51]. This is exemplified by the multitude of attempts to analyse the evolution of complex systems of diverse natures from a Darwinist perspective. It thus follows that the solution to the problem of the origin of life can only be provided on an interdisciplinary basis, with the requisite philosophical background.
– It is demonstrated that the solution to the problem of the origin of life is unfeasible without considering the primary attribute of evolutionary processes, namely, contradiction (in the philosophical interpretation of this term). The aforementioned contradictions can be resolved by employing the methods of dialectics, with illustrative examples drawn from the field of physical chemistry of polymers. This is consistent with the view that evolutionary processes can be understood as a co-evolution of the material and informational 'components' of evolving entities. To substantiate this thesis, the article includes a section titled *The Dialectics of Information.*
– It is demonstrated that the coevolution of the aforementioned type is contingent upon the conversion of a complex system of an arbitrary nature into an analogue of a neural network. The most evident illustrations of this stance are provided by neural networks that emerge spontaneously in solutions of hydrophilic polymers. It is this factor that renders the study of hydrophilic polymers of paramount importance from the perspective of the general theory of evolutionary processes.

## 2. The contradictory nature of understanding evolutionary mechanisms: Preliminary methodological remarks

The analysis of the literature data allows us to say with certainty that two points of view on the nature of evolutionary processes, presumably leading to the origin of life, have been formed to date. One of them, with a certain degree of conventionality, can be called traditional or Darwinist. It is widely represented in the literature, the corresponding review is presented, including in [1]. If we proceed from the general methodological positions, this point of view proceeds from the fact that the primary (from the point of view of the essence of evolutionary processes) are transformations of individual elements of the system (mutations, fluctuations).

The point of view of Vanchurin and his co-authors [43–45] is de facto opposite. In fact, he argues that it is the system properties that are primary, which, with respect to the problem under consideration, are expressed in the fact that the evolving system becomes an analogue of a neural network.

The viewpoint of Vanchurin and his co-authors is not widely accepted; however, there are numerous examples demonstrating that the systems of highly diverse nature can indeed be transformed into analogues of neural networks. In particular, in [52] it was shown that the voting procedure in the Academic (or other) Council can be considered in terms of an analogue to a neural network. This analogue is formed by horizontal links between the members of the Council, and if the density of such links is large enough, the decision is de facto made not by the set of members of the Council, but by the neural network analogue formed by them. This simple example highlights many challenges in social choice theory [53,54]. It illustrates that, in many cases, the outcome of a voting procedure is determined not by individual choices but by the emergent neural network structure created by the collective.

Humanity as a whole indeed forms an analogue of a neural network [55], as all interpersonal communication fundamentally reduces to the exchange of signals between neurons located in different brains. As shown in [55], such a network, formed on a planetary scale, can be equated with the *noosphere* as understood by V. I. Vernadsky [56]. The existence of such a neural network also suggests the presence of a supra-individual level of information processing, which, among other things, plays a role in the formation of the *collective unconscious*. In [57], a hypothesis was put forward that human consciousness itself emerged as a result of evolutionary processes occurring precisely at this supra-individual level of information processing.

Consequently, it is indeed possible to consider a mechanism of evolution alternative to those that go back to the Darwinist point of view. According to it, the primary is the transformation of the analogue of the neural network complementary to the system in question. (Such a transformation may correspond only to a change in the architecture of connections between the elements of the system, while the properties of the elements themselves may remain unchanged.) In the next stage of the system’s evolution, ‘preferences’ are given to those components of the system that are more responsive to its new state. It should be noted that the literature contains a large number of works proving that a change in the architecture of connections between the elements of the system can indeed lead to qualitative transformations of the system as a whole.

For example, a significant number of studies have examined the behaviour of complex systems whose elements are assigned extremely simple properties [58,59]. Often, such elements are considered as abstract ‘nodes’ with a single defining characteristic—the ability to form connections with one another [60,61]. Research of this kind has revealed profound analogies in the theoretical descriptions of seemingly disparate systems. For instance, it has become evident that the theoretical framework for modelling the spread of epidemics is closely related to that of rumour propagation [62,63], among other phenomena. Accordingly, a neural network model based on interacting ‘nodes’ can be used as the foundation for such analyses.

From the point of view of classical dialectics (whether in its idealistic or materialistic version), these works serve as another demonstration of Hegel’s law expressing the transition from quantity to quality. The authors understand that a significant proportion of modern scholars have only a limited familiarity with the laws of classical dialectics. Therefore, this article includes a section titled *The Dialectics of Information,* which, among other things, elucidates the meaning of these laws using material that provides evidence for the conclusions presented in this study.

The authors also realize that in some academic circles the attitude towards philosophy is, to put it mildly, ambiguous, which is connected, among other things, with objective reasons. In particular, the absurdities abundantly generated by postmodernist philosophy are most clearly revealed in the monograph by Sokal & Bricmont [64]. One cannot, however, fail to realize that the problem of the origin of life, when considered in its entirety, cannot but have an ontological aspect. In many respects, we share Sokal and Bricmont’s point of view [64], however, the inferiority of one of the branches of philosophical thought (postmodern) does not cancel out the fact that there are problems of such a fundamental nature that it is impossible to do without using the tools that have been developed by classical philosophy.

Let us demonstrate the factby starting from the results of [43–45] and also [51,55,57]. From the general methodological point of view, the cited works state that a complex system of this or that nature acquires systemic properties (and even properties determining the nature of evolutionary processes) due to the fact that for one reason or another it is converted into an analogue of a neural network. Moreover, in [45], this conclusion is extended to the universe *in toto*.

A neural network [65,66] is a system capable of processing information. Primarily, neural networks are able to recognize images, which determines the main directions of their practical use [67,68]. Another important property of neural network is their ability to learn [67,68], which is the basis of the concept [43–45].

From our point of view, conclusions of this kind represent more than an important step towards establishing the mechanisms that led to the emergence of Life, as well as the construction of a general theory of the evolution of complex systems [51]. In itself, however, this step does not answer the question formulated above—what are the mechanisms that ensure the spontaneous emergence of information processing systems? It seems obvious that the formation of an analogue of a neural network does lead to the emergence of a new quality (and in the sense of classical dialectics), but it is not entirely clear what exactly this new quality consists of. It is important to emphasize that information processing by a system composed of atoms or molecules represents an entirely new quality—one that cannot, in principle, be inherent to the individual elements of such a system, as they can only engage in physical interactions. However, in order to address the aforementioned questions systematically, it is first necessary to consider the philosophical interpretation of the concept of *information* itself. While this term is widely used in practice, the question of its fundamental nature is far from trivial.

## 3. The dialectics of information

This section serves two interrelated objectives. First, we will demonstrate that the interpretation of the essence of information and informational processes through the lens of classical dialectics [69] is indeed fundamental to understanding evolutionary mechanisms, including those that led to the emergence of life. Second, we will provide a brief overview of the fundamental laws of classical dialectics, contextualized within contemporary material closely linked to the rapid advancement of information technologies.

It is important to emphasize that the interpretation of philosophical laws is inherently different from that of physical laws. Seeking to validate their adequacy with mathematical precision would be an oversimplification. The laws of dialectics are considered the broadest possible generalizations of patterns derived from specific scientific disciplines. The validity of this approach has been substantiated through numerous concrete examples, as argued in [70]. The material presented below offers additional evidence in support of this perspective.

### 3.1. The essence of information and the Law of Unity and Struggle of Opposites

The Law of Unity and Struggle of Opposites is one of the three fundamental laws of Hegelian dialectics. As Engels pointed out in [70], the manifestations of this law are highly diverse. The relationship between the material and informational aspects of existence also conforms to this law. Indeed, even the simplest considerations suggest that the properties of information are fundamentally opposed to the properties of matter (the term is placed in quotation marks because, in philosophical literature, the term ‘attribute’ is typically used in such contexts).

For example, information can be copied an unlimited number of times. An informational object, such as a computer program, once created, can be replicated infinitely. In contrast, any object in the physical world exhibits a clear unity between its material and informational components [69]. In the simplest case, any object at the very least contains information not only about itself but also about other objects similar to it. For instance, by studying specific samples of a polymer solution, one can obtain information about the polymer in general (i.e. independently of any specific instance).

These considerations may seem trivial, yet they provide a clear illustration of the numerous debates on the nature of information that have been ongoing in the scientific literature for over half a century.

In particular, the literature—especially in [26,71,72]—contains numerous attempts to provide a *definition* of information. However, from our perspective, such attempts are inherently meaningless [69]. To give a definition (in the ‘school’ sense of the term) means to reveal the meaning of some word using other words. Consequently, there must exist concepts in a language that cannot be disclosed in this way. Otherwise, a logical vicious circle arises. The way out of the situation can be found using the tools of objective dialectics. It asserts the existence of fundamental (indefinable) concepts, particularly philosophical categories, which cannot be explained using the conventional ‘textbook’ definition approach.

They have no definition in the above sense. The essence of philosophical categories is revealed through opposition (e.g. the category ‘Content’ is opposed to the category ‘Form’).

From our point of view, information should be treated as a dialectical category, paired with the category of matter [69]. We emphasize that this opposition reflects not only the challenges encountered in attempts to interpret the essence of information but also an objectively existing reality. Matter and information, as opposites, manifest themselves precisely in their unity—in full accordance with the Hegelian philosophical law under consideration.

This conclusion, among other things, allows us to speak about the existence of a hierarchy of information objects. Just as there is an infinite variety of forms of ‘matter’, so there is an infinite variety of forms of ‘information’, more precisely information objects [69].

The rules of addition of decimal or binary numbers can themselves be considered as a message, i.e. as a sequence of alphanumeric symbols, the amount of information in which can be measured according to Shannon’s formula [73]. At the same time, they can be used to obtain new information, i.e. the result of addition. Consequently, addition rules represent a qualitatively different information object than a message about a particular fact [69].

A similar conclusion is also true for such information objects as geometry theorems, Newton’s mechanics formulas, etc. They also allow us to obtain new information, for example, to calculate the characteristics of an object that is still at the design stage. Therefore, they are also qualitatively different information objects.

This observation, along with similar ones, allows us to formulate the principle of dialectical symmetry [69], according to which there exists a well-defined hierarchy of informational objects—one that closely parallels the hierarchy introduced when considering only the material aspect of objective reality.

At the lowest level of this informational hierarchy lie simple messages, encoded, for example, in alphanumeric form (possibilities related to chemical encoding will be discussed later). At the highest level of this hierarchy is human consciousness. Notably, as emphasized in [57], human consciousness is an object of purely informational nature. Strictly speaking, it does not physically exist; what physically exists is only the exchange of electrochemical impulses between neurons in the brain.

More precisely, as material objects progress from lower to higher levels along the evolutionary scale, they correspond to increasingly complex informational objects.

For example, relatively simple informational objects such as computer programs (which, despite their complexity, still occupy a relatively low position in this hierarchy, as they merely generate new information) correspond to objects such as viruses, which occupy an intermediate position between living and non-living matter. From the perspective of physical chemistry, a virus represents an interpolymer complex that executes a single program—self-replication.

Furthermore, the principle of dialectical symmetry [69] demonstrates the constructive nature of the approach in which information is regarded as a dialectical category, paired with the category of matter.

There is, however, an important nuance. The opposition between the categories of matter and information does not, by itself, provide an answer to how exactly the concept of information should be interpreted in relation to practical tasks. In practice, information is generally used in a form that holds some degree of value.

Attempts have been made in the literature to introduce the concept of valuable information [26], but these efforts have inevitably encountered insurmountable difficulties. For instance, information contained in a mathematics textbook for second-year university students is inherently useless to a large segment of young people who have long since forgotten even their high school mathematics. Likewise, it holds no value for a professional mathematician. This simple reasoning demonstrates that the value of information is inherently dependent on the recipient, making it impossible to assign an objective meaning to this concept.

Another attempt to refine the notion of valuable information is found in the concept of macroscopic information, as introduced in [74,75]. The rationale behind this concept is straightforward: any macroscopic object, such as a polymer solution, can theoretically be characterized in extreme detail—describing the precise arrangement of macromolecular chains, their conformation at every moment in time, etc. However, representing such exhaustive information is clearly meaningless, as all these characteristics change over time. The only valuable information in this case is that which is expressed through macroscopic parameters, such as thermodynamic variables.

Nevertheless, the applicability of this approach is inherently limited. Specifically, for it to be truly viable, the system under consideration must conform to certain statistical regularities (e.g. those established through statistical mechanics).

The methods of objective dialectics allow for overcoming the aforementioned difficulties. The key tool for this is the concept of alienated information [76], which is closely related to another Hegelian law—the law of negation of negation.

The category of alienated information enables, among other things, a deeper understanding of the category of a signal. In simplified terms, an interaction (some physical process) becomes a signal when it either facilitates the transfer of alienated (i.e. unrelated to itself) information or supports the process of information alienation [51].

In the educational literature on the theory of electrical communication, a signal is interpreted as a physical process that ensures the transmission of information [77,78]. With respect to the problems under consideration, however, this interpretation needs significant clarification. If we are talking about the use of a telegraph line, there is no question about what information is being transmitted (and what exactly it is). In this case, intuitive perceptions are sufficient. If we are talking about processes in hydrophilic polymer solutions, the distinction between a ‘simple’ interaction and a process that provides information transfer is not obvious. In particular, if we proceed from the assumption that the formation of information objects in hydrophilic polymer solutions is the key to understanding the mechanisms that led to the emergence of Life, then it is the question of information exchange in such systems that comes to the fore. These considerations necessitate examining the category of a signal from a general methodological perspective [51], which, in turn, brings us back to Hegel’s Law of Negation of Negation.

### 3.2. The Law of Negation of Negation: the category of alienated information and the category of signal

The Law of Negation of Negation—another fundamental law of Hegelian dialectics—can be formulated as the triad ‘Thesis—Antithesis—Synthesis’. This triadic structure can be conveniently illustrated using a classic example from the foundations of quantum mechanics.

Newton’s corpuscular theory of light (which corresponds to the Thesis in this triad) was later challenged and replaced by an opposing viewpoint—the wave theory of light. In philosophical terms, this corresponds to the negation of the Thesis, i.e. the transition to the Antithesis. Finally, modern physics reconciled these opposing views in the form of the wave-particle duality of light, which corresponds to the Synthesis in the triad.

Now, let us demonstrate how this law allows us to overcome the conceptual difficulties associated with the intuitive understanding of the ‘value’ of information, as discussed in §3.1.

The reasoning is as follows. As previously emphasized, the nature of any real object is dual—it possesses both material and informational components (at the very least, any object ‘contains’ information about itself). These components are dialectically interconnected; however, there exist conditions under which this connection is disrupted. A simple example is the experimental measurement of an object’s parameters—for instance, a polymer solution. In this case, information is alienated from the object, acquiring a distinct quality.

According to the Law of Negation of Negation, the Thesis in this case represents the dialectical unity between the material and informational aspects of a given object. The destruction of this unity—that is, the very act of alienating information—corresponds to Hegel’s Antithesis.

Subsequently, in accordance with the law under discussion, the Thesis and Antithesis, as opposing yet interrelated forces, must give rise to a Synthesis. By systematically applying the framework of objective dialectics [51], we ultimately arrive at the dialectical category of a signal.

**Figure 2.**
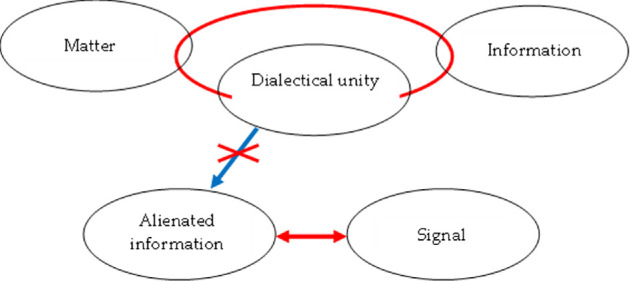
Towards a treatment of the category of signal.

The interpretation of ‘signal’ as a dialectical category also aligns with the concept of Synthesis as the unity of Thesis and Antithesis in their dialectical opposition. This construction is illustrated in figure 2.

Indeed, a signal corresponds both to the Thesis (as it carries information about a real object, understood as a dialectical unity of material and informational components) and to the Antithesis (as it is formed as a result of the disruption of this dialectical unity).

At first glance, the statements presented above may appear purely abstract. However, they provide the foundation for understanding the informational nature of evolutionary processes, including those that ultimately led to the emergence of life.

More specifically, this perspective allows us to determine at what stage signals first appear in physicochemical systems—that is, when this new quality arises, leading to the formation of objects of a purely informational nature, capable of initiating more complex evolutionary processes.

This question will be examined in the following sections. For now, however, let us consider the interpretation of another law of classical dialectics in its application to contemporary challenges related to the development of information technologies.

— *The Law of the Transformation of Quantity into Quality: Neural Networks and Natural Languages*

Numerous examples from chemistry demonstrating the validity of this law—and, more precisely, its universal nature—were presented as early as [70]. This Engelsian law of classical dialectics [70] also aligns closely with the principles of systems theory, originating from the ideas of von Bertalanffy [79], one of which can be formulated as follows:

A system is something qualitatively different from the mere sum of its constituent elements.

An illustrative example is neural networks, where the emergence of new qualities (in the philosophical sense of the term) is directly linked to the number of elements. A small group of connected neurons cannot constitute a fully functional network; instead, new properties emerge only when the number of elements reaches a critical threshold. Even more strikingly, the transition from quantity to quality becomes evident as the connectivity of the neural network increases, a phenomenon confirmed by mathematical models [80].

From the perspective of this study, another particularly relevant informational object is the symbol, as its philosophical interpretation is also significant in the context of the origin of life. In simplified terms, the nucleotides of DNA and RNA, which encode biological information, can also be understood as symbols, provided that we consider Life as a unity of material and informational components.

However, the question of algorithmizing natural languages, which has gained prominence due to advances in machine translation [81], compels us to view this issue from a somewhat different perspective [82]. The cited study clearly demonstrated that the meaning of words in a natural language arises from the connections between concepts, which correspond to specific sequences of letters or sounds. These sequences do not inherently possess meaning; rather, meaning emerges only because a particular word is integrated into a well-defined system.

This consideration may seem self-evident. Moreover, it fundamentally underpins research in ontology (in the technical sense of the term) [83]. However, a far less obvious conclusion was also drawn in [82].

Under certain conditions, the system of concepts that constitutes a natural language acquires the ability to evolve—meaning that it becomes an informational object in its own right, capable of generating previously non-existent informational objects.

For example, a new term (a novel sequence of letters) introduced in a scientific paper gains meaning through its interpretation in relation to existing terms.

At first glance, this idea may also appear trivial. However, a crucial distinction must be made:

Different systems of interconnected symbols exhibit varying degrees of transformation. Some of these systems evolve into full-fledged information processing structures of the type described above, while others do not.

In summary, it is reasonable to assert that the hierarchy of informational objects discussed earlier is itself shaped by systemic factors—which, in turn, can be interpreted through the lens of the philosophical law of the transformation of quantity into quality.

## 4. The evolution of complex systems as the co-evolution of the material and informational components of objective reality

The conclusions drawn above bring us back to the fundamental question raised in [26]: Evolution, which ultimately led to the emergence of Life, can be understood as a continuous process of forming carriers of increasingly complex informational objects.

It is important to emphasize that, from the perspective of the dialectics of information, we must distinguish between the carrier of an informational object and the informational object itself.

The pair ‘informational object carrier—informational object’ can be examined through the lens of the philosophical law of unity and struggle of opposites.

With a certain degree of convention, we can also distinguish two ‘levels’ of organization in any system that acts as a carrier of an informational object: ‘The element level’ and ‘The system level’.

Based on this distinction, the mechanisms of evolution of complex systems of various natures, discussed earlier, can be classified as follows:

— Evolution from the ‘element level’ to the ‘system level’.
— Evolution from the ‘system level’ to the ‘element level’.

Any evolutionary mechanisms that align with the Darwinian perspective clearly correspond to the first of these two variants—that is, evolution proceeding from the element level to the system level. Conversely, Vanchurin’s viewpoint corresponds to the second variant, as it essentially posits that evolutionary processes occurring in the ‘material’ world are directly influenced by informational objects. As noted earlier, in one case, the primary driver of evolutionary processes is the behaviour of system elements. In the other case, it is the behaviour of the system as a whole that takes precedence. This opposition (evolutionary change driven by elements versus evolutionary change driven by system properties) allows—at least formally—for the application of the classical law of unity and struggle of opposites.

A direct application of this law suggests that the nature of evolutionary processes is simultaneously determined by both systemic properties (particularly neural network properties) and the ability of elements to change their properties (e.g. through adaptation to environmental conditions, which was the foundation of Darwinian theory). Depending on the specific context, one or the other of these two facets may dominate.

**Figure 3.**
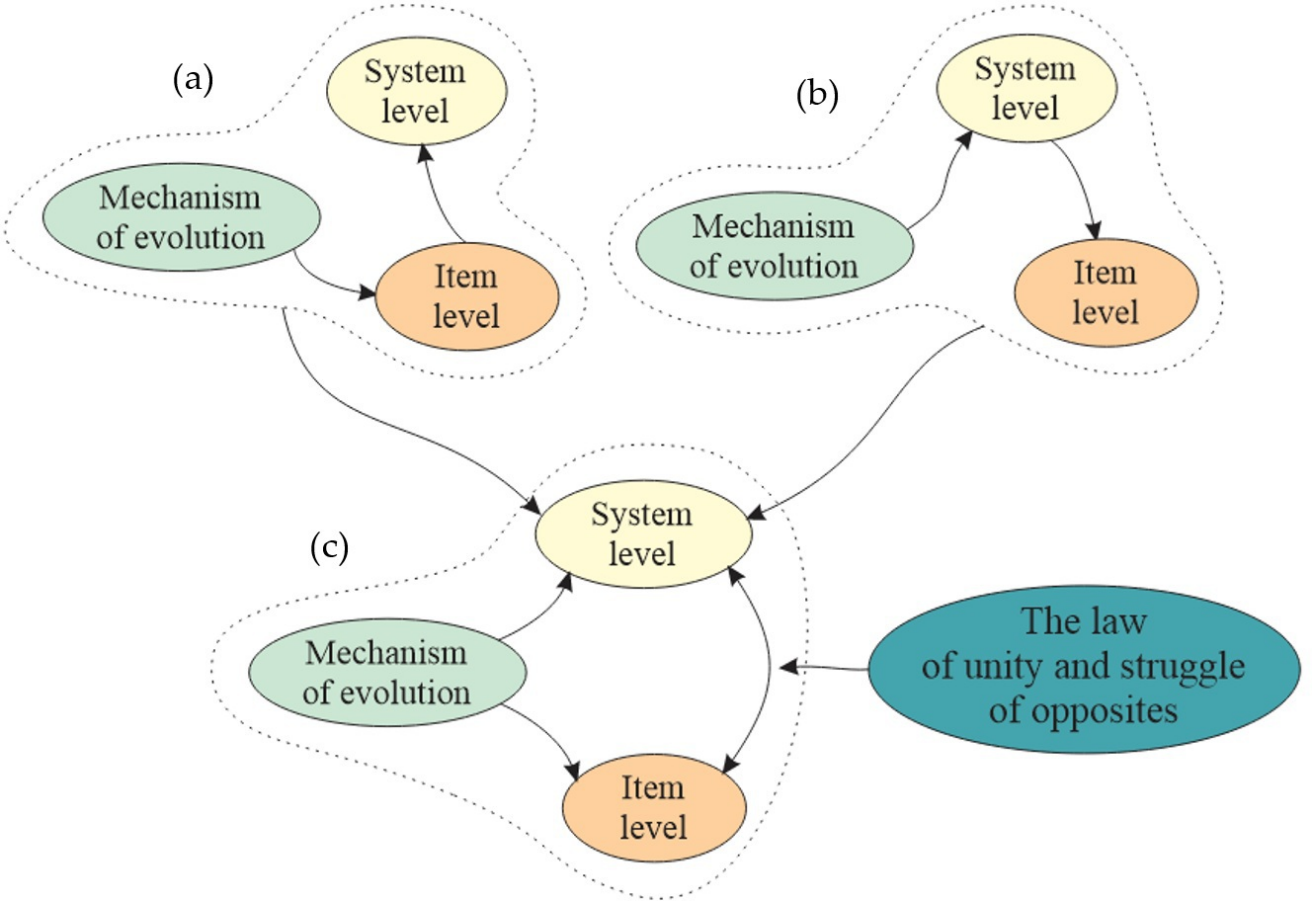
Formal application of the law of unity and struggle of opposites to the interpretation of mechanisms of evolution of complex systems: (a) classical Darwinism; (b) ‘neural network’ approach; and (c) their dialectical synthesis.

A schematic representation of the formal application of dialectical laws to the problem of the evolution of complex systems is presented in figure 3.

Evolution—including that which led to the emergence of Life—is in fact a co-evolution of objects, which with a certain degree of convention can be called informational (because they cannot exist without a carrier), as well as objects of material (in the prosaic sense of the term) nature, which, among other things, can act as a carrier of informational objects.

Simplifying, evolution is generated by the dialectic of interaction between the ‘informational’ and ‘material’ facets of being. This thesis fully corresponds to the concept of dialectical positivism first proposed in [84], which, in particular, attempts to remove the contradictions between materialism and idealism. A detailed discussion of these issues, however, is beyond the purpose of this review. The authors hope that the considerations presented above are sufficient to justify the use of the tools of classical dialectics in discussing the problem of the origin of Life (as a special case of the general theory of evolution) from the point of view of the physical chemistry of polymers.

It is therefore evident that information processes occurring in systems based on hydrophilic polymers are of paramount importance. The transfer of information, as exemplified by the behaviour of DNA and RNA, is an inevitable consequence of the long evolutionary history of these systems. Consequently, it is crucial to determine at what stage, and due to what, information properties are acquired by simpler polymers.

It should be emphasized that everything that makes any **analogue of a neural network** a distinct **actor** in the evolutionary process can also be of a purely **informational nature**. Like consciousness, which is formed by the exchange of signals, a physically existing analogue of a neural network can arise only as a result of interaction between the elements of the system, which objectively must be converted into the exchange of signals. Otherwise, a new informational quality cannot be formed.

Here arises the fundamental—mainly philosophical—question of what exactly distinguishes a simple ‘interaction’ (e.g. the formation of an ionic bond between the functional groups of polymers or the exchange of electrochemical impulses between neurons in the brain) from a signal, i.e. from some entity that is *capable of carrying information* [51].

Paradoxically, the most illustrative answer to this question—even from a purely applied perspective—can be found in the existing literature on hydrophilic polymer research, which directly aligns with one of the main objectives of this review.

The proof of this statement is provided in the following sections.

## 5. The category of alienated information and prototypes of evolutionary processes in the physical chemistry of polymers

As demonstrated above, the simplest form of an informational object, without which no information processing system can function, is alienated information. Consequently, the initiation of any evolutionary processes that lead to the emergence of informational objects can only be driven by physical or physicochemical interactions in which information alienation occurs.

**Figure 4.**
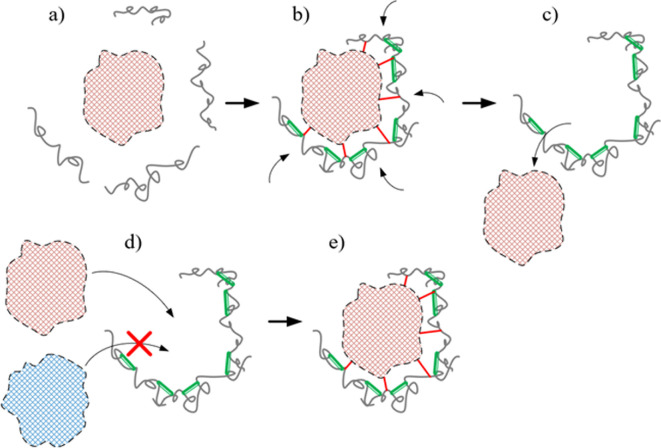
Mechanism of formation of tunable sorbents, simplified scheme (a) object and polymer chain before interaction, (b) result of interaction of object and polymer chains, (c) formation of a system capable of recognising an object, (d) condition for recognising an object, (e) result of recognising an object.

Processes that can be interpreted as the simplest form of information alienation have long been known in physical polymer chemistry. In particular, tunable sorbents have been well studied [85–87], the mechanism of which is simplistically illustrated in figure 4.

Initially, polymers capable of forming a matrix are brought into interaction with an object that either coincides with the objects to be recognized or is close to them in properties (figure 4a). Next, macromolecules are cross-linked to form a structure complementary to the used object (figure 4b). In the next step, this object is removed in one way or another (figure 4c), resulting in the formation of a customizable sorbent. The obtained structure turns out to be able to ‘recognize’ objects that are close in characteristics to the original one (figure 4d,e). It can be seen that in this case there is indeed a form of information alienation: the structure of the tunable sorbent carries information not only about itself, but also about the object that was used for its synthesis.

From the same point of view, it is acceptable to consider sorption processes occurring with the participation of inorganic materials with a large surface area (porous minerals, nanoparticles, etc.). The specific distribution of electrostatic charges and the topology of the sorbent surface can set quite a certain arrangement of adsorbed molecules on its surface. Provided that the sorbed molecules (or macromolecules) further enter into interactions that fix their arrangement set by the specificity of the sorbent, the surface of the latter acts as a matrix.

Examples of nontrivial orientation effects involving substances sorbed by clays and minerals have been known for quite a long time [88,89]. It is also known that minerals such as montmorillonite are capable of creating favourable conditions for polymerization and assembly of molecules [90,91], and the cited works attempted to use such effects to establish mechanisms of prebiological evolution.

The presence of some ‘template’ or matrix, at least theoretically, can lead to the accumulation of similar molecules over time. Such accumulation may contribute to reaching a critical threshold when self-organization becomes spontaneous, which is a key step towards the formation of increasingly complex molecular systems (the concept of ‘critical mass’ [92,93]). Replication processes, where surfaces act as templates for the assembly of biomolecules, have also been considered in [94–97] as the basis of mechanisms for the emergence of early life-like systems such as RNA precursors. Sorbents could play an active role in the formation of early replicative systems by sorting and concentrating nucleotide building blocks, thus being a prototype of proto-RNA systems.

**Figure 5.**
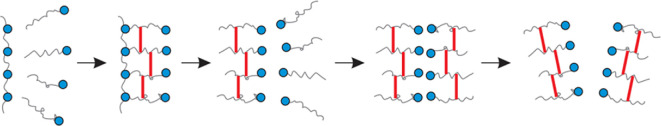
Illustration of possible replication mechanisms, the ‘start’ of which is set by the specificity of the sorbent surface.

A simplified scheme summarizing the mechanisms discussed above is shown in figure 5. This scheme emphasizes that the configuration of the supramolecular system, initially set by the specificity of the sorbent surface, under certain conditions can be further reproduced by interactions in which the sorbent does not participate.

As noted in the works cited above, the ability of sorbents to reproduce molecular configurations may reflect the processes observed later in RNA replication, thus providing a model for the origin of early informational macromolecules. It can be seen that in this case, too, there is a process of information alienation, moreover, this information is reproduced repeatedly (it is ‘overwritten’).

Note also that the role of the sorbent (more precisely, the role of the matrix), schematically depicted in figure 5, does not necessarily have to be inorganic substances. A relevant example is the phosphate backbone of nucleic acids, which actively interacts with proteins, ions and other biomolecules, causing molecular recognition and binding [98,99]. Cross-linked polymer networks (hydrogels) can also play such a role. Moreover, it is polymer networks that can potentially be considered as some kind of matrix arising in the process of self-organization. However, in order to analyse this possibility, it is necessary to consider the neural network properties of systems formed in hydrophilic polymer solutions.

It is emphasized that it is the neural networks based on hydrophilic polymers that align with the concept reflected in §3. Indeed, on the one hand, they align with the approach put forth by Vanchurin [43–45], as they effectively introduce a neural network into the system, which is a crucial element in the aforementioned approach. Conversely, the behaviour of the object is contingent upon the potential transformations of its constituent elements, which may be considered analogous to neurons.

Furthermore, neural networks based on hydrophilic polymers exemplify a system that can evolve holistically, whereby the neural network undergoes rearrangement while the properties of individual elements remain unaltered. This situation is fundamentally distinct from the reduplication-based evolutionary schemes discussed in the aforementioned papers. It is possible for evolution to proceed not from an element to a whole, but from a whole to an element. In an initially simple system, as it evolves, more and more complex components emerge. As previously noted, this can be regarded as a process of self-learning for the network.

## 6. Formation of neural network analogues in hydrophilic polymer solutions

**Figure 6.**
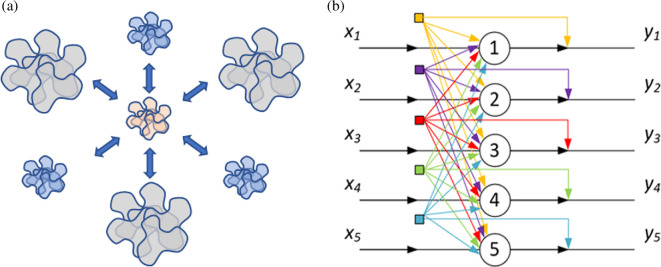
Generalized scheme of neural network analogue formation in hydrophilic polymer solutions (a) Hopfield neural processor scheme (b) [41].

The possibility of spontaneous formation of a neural network analogue in a solution containing hydrophilic polymers was demonstrated in [41] using a specific example. The scheme of neural network analogue formation proposed in the cited work can be represented in the following generalized form (figure 6a). According to this scheme, a single macromolecular coil in aqueous solution, capable of experiencing a phase transition, is considered as an analogue of a single neuron. It is assumed that the phase transition corresponds to a jump-like change in the degree of swelling of the coil (from maximally swollen to a state close to loss of solubility). The swollen state corresponds to a logical unit and vice versa. It is also assumed that all the coils are able to influence each other, which allows us to compare the considered scheme with the classical scheme of the Hopfield neural processor [100] (figure 6b). It can be seen that these two schemes are topologically equivalent.

This generalized scheme is based on the following facts, repeatedly reflected in works on the physical chemistry of hydrophilic polymers:

— There are varieties of macromolecules that experience a phase transition even at relatively large variations of the controlling thermodynamic variables, these are stimulus-sensitive polymers [101–103], an important variety of which are thermosensitive polymers [104–106].
— Provided that the macromolecules experiencing the phase transition have dissociating functional groups in their composition, the nature of the phase transition is determined, among other things, by the ionic strength or acidity of the solution [107–109].
— There is a concentration redistribution effect directly detectable for the case of cross-linked polyelectrolyte networks [110–112]. This effect is entirely due to the fact that thermodynamic equilibrium is established between the volume occupied by the polyelectrolyte mesh and the surrounding solution. Due to the fact that the concentration of counterions in the mesh volume should balance the electrostatic charge of its functional groups, the concentration of low molecular weight salt inside the polyelectrolyte hydrogel sample will always exceed its concentration in the surrounding solution, which can be used for technical purposes [111,112]. This effect is also evident for macromolecular coils when their size significantly exceeds the Debye length.
— The nature of the phase transition of the ‘basic’ polymer can be influenced by the presence in the solution of even relatively small concentrations of the ‘additional’ polymer, which can lead to the formation of an interpolymer complex [113–115]. The same is true for the presence of polyvalent metal ions in solution [116–118].

**Figure 7.**
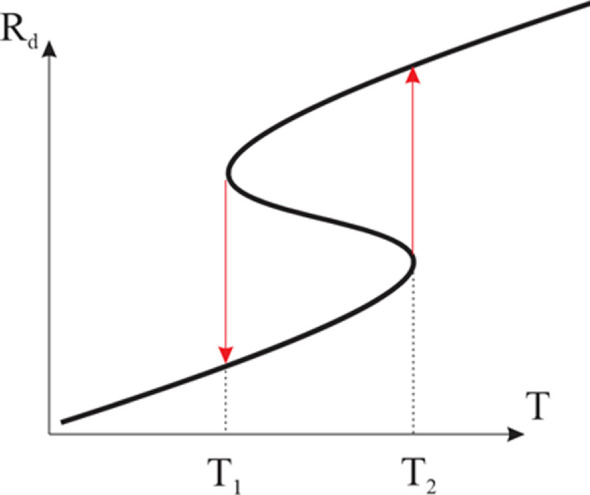
To the mechanism of hysteresis phenomena at phase transitions.

The above effects lead to the fact that there are conditions when the polymer solution in which the phase transition takes place is no longer homogeneous. Some macromolecules may experience a phase transition, while others may not. We emphasize that the existing theories of phase transitions usually lead to the conclusion that it is discontinuous [119,120]. This conclusion is clearly illustrated in figure 7. This figure shows an S-shaped curve that describes the dependence of the recorded parameter (e.g. the hydrodynamic radius of a macromolecular coil) on the control parameter (e.g. temperature). The arrows in this figure represent the temperatures at which the forward and reverse phase transition takes place.

It can be seen that if the phase transition really corresponds to the transition from a non-monotonic dependence to a non-monotonic one (S-shaped curve), then the phase transition should not only be discontinuous, but also be accompanied by hysteresis phenomena. Such phenomena can indeed sometimes be detected [121], however, in reality, the temperature range in which the phase transition occurs is quite extended [122–124]. From our point of view, this is obviously related to the inhomogeneity of the solution. At least, as the data of [125,126] show, the phase transition temperature can depend significantly on the molecular weight of the polymer. Consequently, the situation when some macromolecules have experienced a phase transition and others have not is quite realistic.

The heterogeneity of hydrophilic polymer solutions is a prerequisite for their consideration in terms of analogy with neural networks. Indeed, the possibility of a phase transition of a single macromolecular coil allows us to assign a logical 0 to 1 of the states shared by such a transition and a logical one to the other.

To substantiate the neural network analogy, following [41], it remains to make the last step, i.e. to demonstrate that neuron analogues (macromolecular coils) are indeed able to influence each other.

**Figure 8.**
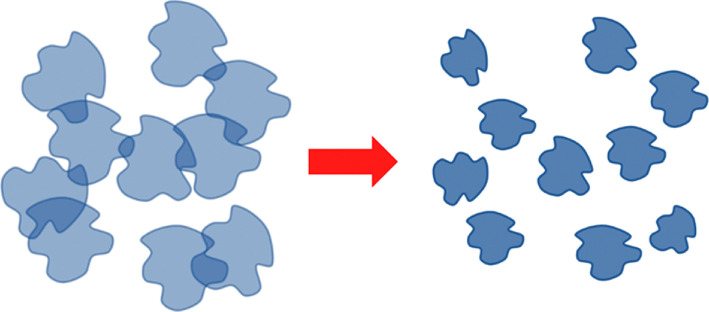
Schematic of the direct interaction between macromolecular tubules (a case of HHA formation [42]).

The simplest form of such interaction is the direct interaction of coils through the formation of hydrogen bonds, hydrophobic interactions, etc. An example is hydrophobic interactions that lead to the formation of hydrophobic–hydrophilic associates (figure 8) [42]. In this case, it is the hydrophobic interactions between clubs that lead to the emergence of a mesh existing in a dynamic regime (the bonds between the clubs are continuously formed and destroyed again).

**Figure 9.**
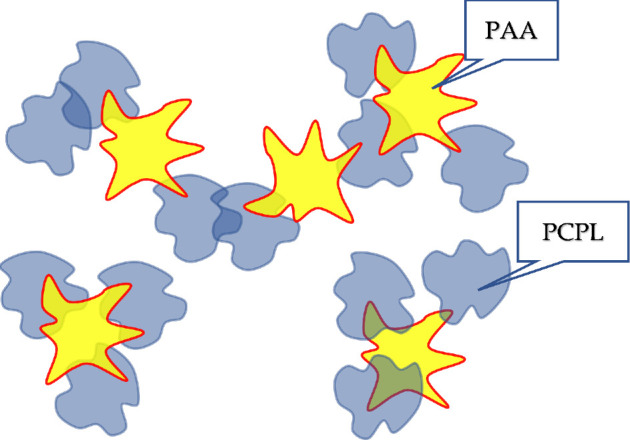
Schematic of the direct interaction between macromolecular tubules (case of simultaneous formation of HHA and HIA [41]); PCPL, polyvinylcaprolactam; PAA, polyacrylic acid.

Direct interactions can lead to the formation of more complex systems, in particular, when there are two types of macromolecules in the system. In such cases, polymer networks can also arise, but already stabilized by both hydrophobic and hydrophilic interactions (figure 9) [42].

Evidence for the existence of such associates is also given in [127–129]. The same factors also lead to the formation of an analogue of a neural network [41]: an example of the corresponding mathematical model would also be presented in the cited paper.

Further, the interaction between macromolecular coils, provided that they have a sufficiently high degree of ionization, may be due to a concentration redistribution effect [110]. If an individual polyelectrolyte coil experiences a phase transition, this will (albeit to a small extent) affect all others. Indeed, in the swollen state, a polyelectrolyte ball placed in a water–salt solution accumulates predominantly water, displacing salt into the surrounding solution. If the coil shrinks, the salt concentration in the surrounding solution decreases (especially if the dissociation of the functional groups of the coil is suppressed). Variations in the ionic strength of the solution, in turn, can influence the degree of swelling of the macromolecular coils due to the well-studied polyelectrolyte effect [130–132]. It is significant that this effect can be enhanced when the macromolecule contains not only dissociating but also hydrophobic functional groups [133,134].

**Figure 10.**
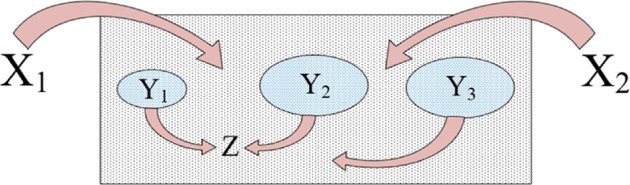
A model example of a neural network analogue formed by three polyelectrolyte clubs placed in an aqueous salt solution.

The example of mediated interaction between macromolecular coils due to the polyelectrolyte effect, as well as the effect of redistribution of concentrations, allows us to present a model example of a neural network based on phase transitions in hydrophilic polymer solutions, which admits a clear mathematical description in terms of logical functions (figure 10) [51].

This model example, constructed using the neural network analysis technique proposed in [135], contains three polyelectrolyte coils (it is labelled with symbols $Y_{i}$), placed in a water–salt solution (it is labelled with the symbol Z). It is assumed that each of these coils is capable of experiencing a phase transition, due to which the salt concentration in the solution changes (due to the concentration redistribution effect). The external influences in this scheme are labelled with the symbols $X_{1,2}$ [112].

**Figure 11.**
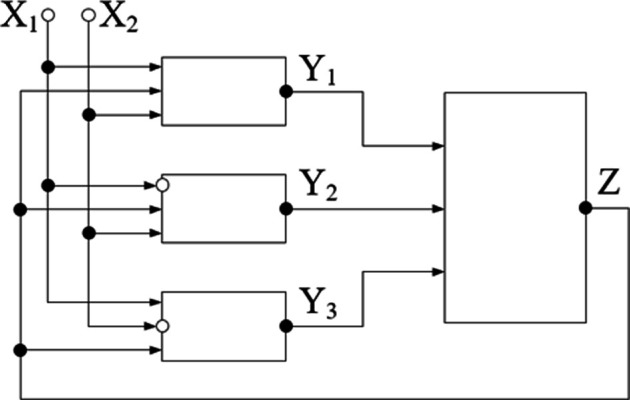
Equivalent logic circuit corresponding to the neural network analogue circuit shown in figure 10.

This example is purely modelling, but it allows us to identify the essential features of neural network analogues that can be formed in hydrophilic polymer solutions. The advantage of this example is also that it allows us to pass to the equivalent circuit built on logic elements (figure 11).

In the circuit of figure 11, each of the elements $Y_{i}$ (neuron analogue) has three inputs. These elements form the first layer of the model neural network. Two inputs correspond to the possibility of using two thermodynamic variables $X_{1,2}$ (e.g. temperature and concentration of low molecular weight salt) to control the degree of swelling of polyelectrolyte tubules. The third input corresponds to the existence of feedback due to the effect of redistribution of concentrations.

The same control signals are fed to the inputs of each element of the first layer of the neural network with numbers 1, 2 and 3, which corresponds to the simultaneous influence of two changing thermodynamic variables on all samples in solution.

The outputs of the neural network elements are connected to a single neuron of the second layer Z which corresponds to the interaction of the elements of the system under consideration through the environment in which they are located. A feedback loop linking the output of the element Z to the inputs of the elements $Y_{i}$ corresponds to the mutual influence of cross-linked polymer meshes according to the above mechanism.

It was shown in [135,136] that if the activation function of a neuron is close to the threshold function (which, with respect to the system under consideration, corresponds to a jump-like change in the degree of swelling of the ball), then the operations performed by the neurons of the network can be reduced to logical functions. In particular, the description of the neurons of the first layer of the network shown in figure 11 can be reduced to logical functions of the following form


(6.1) $$Y_{i}\;=\;\left(X_{1}+a_{1i}\right)\left(X_{2}+a_{2i}\right)\;+\;\left(X_{1}+a_{1i}\right)\left(Z\;+\;a_{3i}\right)\;+\;\left(X_{2}+a_{2i}\right)\;\left(Z\;+\;a_{3i}\right).$$

In this formula, the coefficients $a_{ij}$ can take values either 0 or 1: $a_{ij}=(0,1)$, which corresponds to the situation when individual inputs of the neurons of the first layer are inverse (physically, this corresponds to the opposite character of the reaction of the tangle to temperature change, which is quite realizable, since there are polymers with both upper and lower critical solubility temperature [133]).

The output state of a single neuron of the second layer is described by a similar formula:


(6.2) $$Z\;=\;Y_{1}Y_{2}+Y_{2}Y_{3}+Y_{1}Y_{3}.$$

It is easy to show that for any combination of input variables $X_{1}$ и $X_{2}$ in this logic circuit, the variable Z can acquire the value of both logical 0 and logical 1. In other words, four possible combinations of input variables correspond to eight possible combinations of values of logical variables describing the state of the outputs of the neurons of the first layer. In other words, in this case, all possible eight combinations of logical variables can be realized at the output of the neurons of the first layer.

The considered example, of course, is no more than illustrative. However, it allows us to assert that there are varieties of neural networks capable of capturing quite specific information even when the same signals are fed to all inputs of all neurons of the first layer.

As applied to the systems of the type under consideration, this means that there is a possibility of recording information at the molecular (or supramolecular) level due to macroscopic influences. In particular, the state of outputs of neurons of the first layer of the model network shown in figure 10 does not depend on the current state of thermodynamic variables, but on how exactly they changed in time, i.e. in this case, changes in thermodynamic variables become code sequences.

We emphasize that this example can be viewed from the perspective of the dialectic of evolution: it shows that the ‘system level’ can indeed influence the ‘element level’.

The authors are aware of the fact that the processes of self-organization leading to the emergence of distinct structures occur, as a rule, in open systems far from the equilibrium state [137–139]. Relevant issues have been considered in numerous works, one way or another related to synergetics, and belonging to various sections of physics, for example, in [140,141], however, their consideration within the framework of this review would lead too far away. We focus the reader’s attention only on the very existence of neural network properties of complex systems, and we have to leave aside all those aspects that are related, for example, to the discussion of energy costs for their realization.

For the purposes of this review, another aspect of the problems under consideration is more interesting. Namely, it raises the question of how exactly an analogue of a neural network formed in solution can ‘go into the self-reproduction mode’, and, most importantly, how effective (from the point of view of forming information processing systems) the formation of analogues of neural networks of the considered type can be.

## 7. The problem of information fixation in analogues of neural networks based on hydrophilic polymers from the point of view of dialectics of evolution

The answer to the first of the questions formulated at the end of the previous section is, in fact, already present in the literature. Namely, the conditions under which cross-linked networks (hydrogels) having a wide range of unique properties (sensitivity to external influences, etc. [142,143]) are formed in solutions of hydrophilic polymers are well studied.

There are also hydrogels formed due to physical cross-linking knots, which are formed as a result of the association of hydrophilic polymer segments and can vary depending on polymer concentration, solution composition and temperature [144]. For the purposes pursued, it is essential that at certain concentrations of polymers in solution, the formation of highly ordered structures with physical knots is possible [145–147].

**Figure 12.**
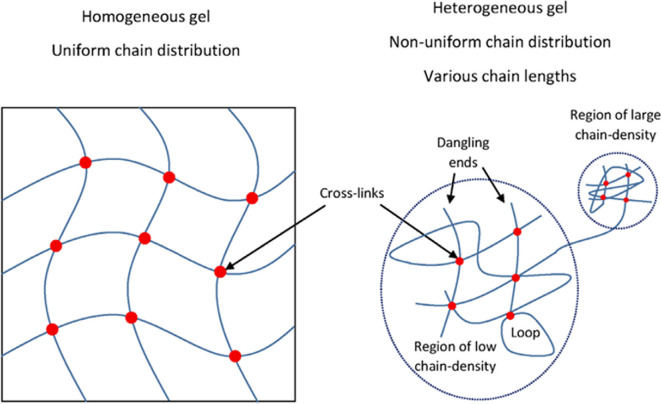
Scheme for the formation of heterogeneous hydrogels due to the formation of inhomogeneous distribution of chains over the volume [148].

It should be noted that the synthesis of inhomogeneous hydrogels has been considered in the literature for a very long time. Thus, in [148] it was shown that hydrogel inhomogeneities can be caused by inhomogeneous distribution of polymer chains over the sample volume, which is associated, among other things, with the formation of loops. This situation is illustrated in figure 12, from the cited work.

**Figure 13.**
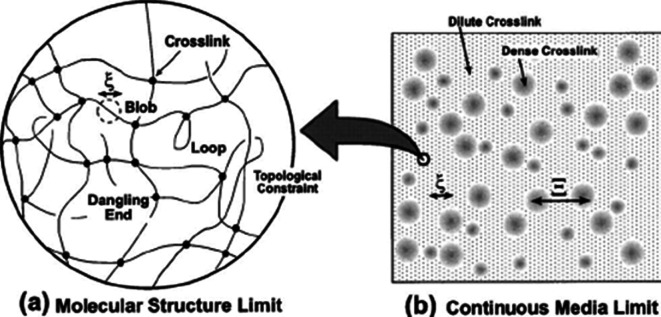
Illustration of the formation of inhomogeneous hydrogels due to inhomogeneous distribution of cross-linking units over the sample volume: (a) Molecular Structure Limit; (b) Continuous Media Limit [149].

**Figure 14.**
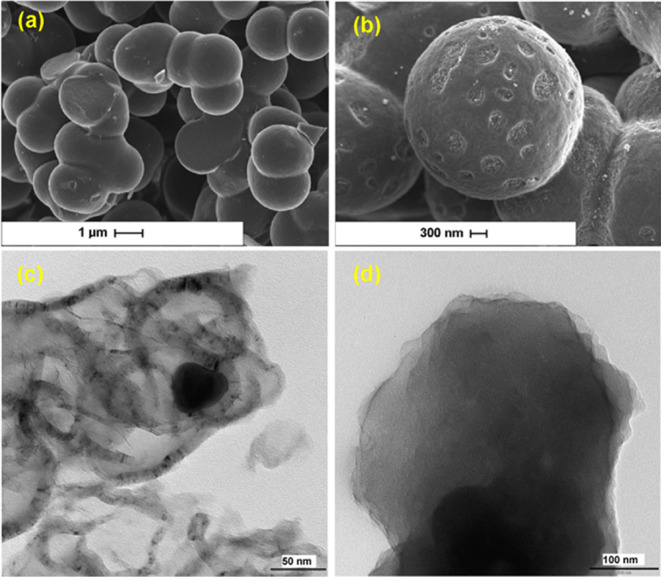
SEM and images of (a) N/S-co-doped and (b) S-doped acid-leached carbon heterogeneous gels. TEM images of (c) N-doped and (d) S-doped leached carbon heterogeneous gels [150].

The factors discussed in [148] are closely related to those noted in [149]: gel heterogeneity may be due to a heterogeneous distribution of cross-linking units over the sample volume (figure 13). It should be emphasized that, in accordance with the conclusions illustrated in figure 13, the heterogeneous distribution of cross-linking units over the gel volume may be due, among other things, to the fact that the gel structure is influenced by the structure of the solution from which it was synthesized. This factor should be particularly evident in cases where the gel is synthesized not on the basis of a monomer mixture, but on the basis of a solution of macromolecules (e.g. by radiation cross-linking). Note also that the cited work presents results directly proving the formation of inhomogeneous hydrogels obtained using the neuron scattering method (figure 14) [149].

There are other works in the current literature that reflect the possibility of obtaining non-nodular hydrogels, notably [151], but for the purposes of this review, further clarification is not as essential. There is every reason to argue that hydrogels with ordered structures represent the simplest example of the formation of systems in which information formed by interactions in solution can be fixed. In addition, in such networks, information can be fixed due to different ways of coding at the molecular level, for example, by changes in the configuration or chemical state of functional groups anchored in the polymer matrix [152,153]. In this case, an analogy can be drawn with the nature of recording and storing information in digital systems [154]. The orderliness of these structures provides an opportunity to create a material capable of long-term storage and processing of information at the molecular level [155]. Hydrogels with an organized grid structure can be adapted for use in information technology and even artificial intelligence systems, where information is encoded through the spatial organization and reactivity of the molecules within the gel [156,157] (it should be noted that this approach is also aligned with current trends towards the utilization of neuromorphic materials [158–160] in electronic devices, specifically for the purpose of direct signal processing). Such a suitable structure for data storage can interact with external signals and change its state in response to stimuli, thus providing the possibility of adaptive information storage and processing systems [161]. Considering the presented examples from a general methodological point of view, we can state that there are conditions for the structure of the neural network analogue of the type considered above to be fixed during gel formation. In fact, for this purpose, it is sufficient only that the structure of the network turns out to be essentially inhomogeneous and its components are able to respond to changes in thermodynamic parameters. In this case, the existence of inhomogeneities (which do not even necessarily have to be ordered) ensures the formation of structures similar to the one shown in figure 6, and, consequently, possessing neural network properties.

The answer to the second question formulated at the end of the previous section is also found in the literature. Distant interaction between macroscopic samples of cross-linked polymer meshes has been found in experiments [162–164]. Moreover, this phenomenon has been proposed to be used for the selective sorption of heavy metal ions [165,166], which is another illustration of the formation of ‘alienated information’ in physicochemical systems based on hydrophilic polymers. Moreover, the effect of remote interaction of cross-linked polymer networks allows us to assert that the systems of the considered type can really realize the transmission of signals to relatively large (compared to the size of macromolecular tangles) distances. The main thing is that this kind of interaction can provide the formation of analogues of neural networks with a sufficiently branched system of connections between elements (which determines the efficiency of a neural network as an information processing system).

The mechanism of remote interaction of hydrogels is as follows [165,166]:

– Let us consider two cross-linked polymer networks placed in a solution of a low molecular weight salt. One of these networks acts as a proton donor (e.g. it can be a network based on cross-linked partially neutralized polyacrylic acid) and the other as a proton acceptor (e.g. it can be a mesh containing amine or imine functional groups).

The simplest example of a reaction occurring in a proton donor mesh is the dissociation reaction of carboxyl groups:


(7.1) $$R_{1}-COOH↔R_{1}-{COO}^{-}+H^{+}.$$

There is an equally simple example of a reaction that takes place in a proton acceptor network:


(7.2) $$R_{2}-{NH}_{2}+H^{+}↔R_{2}-{NH}_{3}^{+}.$$

Taken together, these reactions correspond to the well-known process of proton transfer from the carboxyl group to the amine group:


(7.3) $$R_{1}-COOH+R_{2}-{NH}_{2}↔R_{1}-{COO}^{-}+R_{2}-{NH}_{3}^{+}.$$

**Figure 15.**
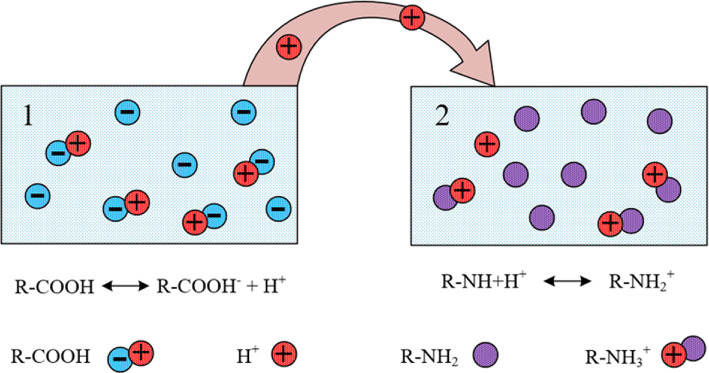
Illustration of the remote interaction of hydrogels.

The specificity of the remote interaction of hydrogels is that in this case radicals $R_{1}$ и $R_{2}$ are rigidly bound to the framework of the corresponding meshes [164,167] (figure 15).

As a result, the pair consisting of a proton donor network and a proton acceptor network performs a kind of ‘cleavage’ of the low molecular weight salt present in the solution above both of these networks. Indeed, the total equation of the reaction occurring at the remote interaction of the considered type can be written in the following form (for certainty, the case when both networks are located in the sodium chloride solution is considered):


(7.4) $$R_{1}-COOH+R_{2}-{NH}_{2}+NaCl↔{\left(R_{1}-{COO}^{-}+{Na}^{+}\right)}_{1}+{\left(R_{2}-{NH}_{3}^{+}+{Cl}^{-}\right)}_{2}.$$

The indices ‘1’ and ‘2’ in the above formula emphasize that the substances concerned and the ions formed by their dissociation are concentrated in different regions of space.

The presented example proves that the analogues of neural networks formed in hydrophilic polymer solutions may possess a very branched system of connections, which, in accordance with the conclusions of [168], is critical from the point of view of the neural network’s ability to process information. It is also significant that neural network analogues can be formed within cross-linked polymer meshes, which may well possess pronounced inhomogeneities. This, as follows from the materials presented above, creates very wide opportunities not only for the formation of very non-trivial information objects but also for their replication. Moreover, interactions related to the formation of inhomogeneous networks and ‘distributed’ chemical reactions (e.g. when the proton donor and acceptor are localized in different parts of the mesh) can also initiate the emergence of more complex processes associated, for example, with the formation of ‘memory cells’ [168]. Note that the hysteresis phenomena found in [121] are themselves responsible for the formation of binary memory cells, since a local element of the system can be in two different relatively stable states.

To conclude this section, we note that the existence of neural network properties inherent in systems based on hydrophilic polymers, especially when considered from the perspective of classical dialectics, removes many methodological difficulties repeatedly voiced by critics of evolutionary theories. In particular, the question of the verifiability of evolutionary theories remains (it goes back to the point of view of one of the recognized experts in the philosophy of science, K. Popper, who once claimed that Darwinism is unverifiable). The use of polyelectrolyte hydrogels as smart materials capable of serving as carriers of non-trivial information objects [168] allows at least partial remove of such objections by creating conditions under which such objects can really evolve. In this regard, it is relevant to note that evolving neural networks are already a subject of study [161,169]. In the future, this creates a basis for setting up appropriate experiments in the ‘accelerated’ time regime.

This issue is not solely of academic interest. Indeed, the experimental implementation of processes that imitate, to some extent, the processes that presumably led to pre-biological evolution creates a number of significant prerequisites, including the development of neuromorphic materials. The necessity for the creation of such materials is indisputably due to the fact that the potential for further development of electronics based on classical binary logic and typical semiconductor elements has been largely exhausted. In particular, in the review [170–172], it was emphasized that the realization of neuromorphic devices/architectures has significant advantages, including ultra-low voltage operation and the ability to form parallel-connected networks with a minimum number of wire connections. These same advantages are reiterated in other works on the subject of neuromorphic materials, in particular in [171]. It is also important to consider the von Neumann architecture, which forms the basis of modern computers. This architecture assumes data exchange between memory blocks and computational blocks. This has a considerable impact on the performance of computing systems of this nature. The transition to a neuromorphic computing paradigm will enable the elimination of the aforementioned disadvantages. It is also noteworthy that a considerable number of publications in the literature attempt to combine computational techniques with the operations performed by biological informational macromolecules [172].

In considering the questions raised by the problem of the origin of life, it is evident that there are two principal aspects to be addressed. One of these considerations is connected with the construction of a general theory of evolution. As previously emphasized, the key problem for which this cannot be the solution is the problem of the origin of life. It is our contention that the creation of such a theory is contingent upon the establishment of a robust philosophical foundation. The applied aspect is related to the fact that even partial establishment of evolutionary mechanisms, which correspond to the mechanisms of pre-biological evolution to a greater or lesser extent, makes it possible in the future to proceed to the creation of various types of quasi-biological computing systems, and subsequently to other devices built on the principles of 'quasi-life'.

## 8. Conclusion

Thus, there are various prerequisites for considering the problem of the origin of life in the most generalized form. Evolution, which led to the appearance of living organisms, is inextricably linked with the emergence and subsequent transformation of information objects, which form a quite definite hierarchy. Accordingly, the problem of the origin of life can be considered from the standpoint of spontaneous emergence of systems capable of processing information, first in a maximally simple and then in an increasingly complex way.

From this point of view, systems based on hydrophilic polymers in which analogues of neural networks are spontaneously formed deserve close attention. The existence of such systems can serve as a direct confirmation of Vachurin’s hypotheses, according to which neural network properties of complex systems are one of the main factors determining the nature of their evolution. Analogues of neural networks, including those formed naturally, are capable of generating information objects capable of influencing the behaviour of the system as a whole.

It is essential that in the current literature devoted to the physical chemistry of hydrophilic polymers, a sufficient amount of data has already been accumulated which allows us to consider such systems from the information point of view. The authors understand that these data need further systematization and generalization, however, even the data presented in this review are sufficient to confirm the inference arising from general methodological considerations. The problem of the origin of life should be considered from the position of co-evolution of information objects and their carriers. The neural network approach to the interpretation of the evolution of complex systems can be opposed to the ideas going back to Darwinism, which reflects quite certain contradictions objectively inherent in evolutionary processes. This contradiction, however, is resolved through consideration of the dialectical unity of material and informational objects.

This approach is fully consistent with classical dialectics. It returns to the thesis that such fundamental problems as the problem of the origin of life cannot be solved without using the tools of classical dialectics which are purposefully created to deal with contradictions.

## Data Availability

This article has no additional data.
